# O26 More than meets the eye … When inflammation extends beyond the anterior chamber

**DOI:** 10.1093/rap/rkab067.025

**Published:** 2021-10-19

**Authors:** Charlene Foley, Kate Armon, Filippos Skarmoustsos, Brinda Muthusamy, Peter Bale

**Affiliations:** 1 Addenbrooke's Hospital, Cambridge, United Kingdom; 2 West Suffolk Hospital, Suffolk, United Kingdom

## Abstract

**Case report - Introduction:**

The differential diagnosis of paediatric uveitis is extensive. Classification starts by determining infectious versus non-infectious causes, anatomic location and associated intra-ocular and extra-ocular features. A relatively common referral to Paediatric Rheumatology from our Ophthalmology colleagues is of a child with a diagnosis of uveitis.

This case highlights the importance and benefits of multidisciplinary team working across our regional network when caring for children with complex and rare conditions.

**Case report - Case description:**

An 8-year-old-girl was referred by her local ophthalmology team to the paediatric rheumatology clinic with a diagnosis of pars-planitis. She had presented to them with blurred-vision and eye-“floaters”.

On review, the girl reported that she had a 4-month-history of headaches and blurred-vision, associated with dizziness. She denied any eye-pain. Corresponding to the onset of her symptoms, she had suffered with Chickenpox. Her mother felt that she had "not been right since then". She noted that she was generally quieter and more fatigued than normal.

Over the course of the 4 months, she was noted to have become clumsier and was bumping into things on a regular basis. She reported increasing visual difficulties in her right eye and initially attended for an optician review. The optician was concerned and referred for urgent ophthalmology opinion. She was diagnosed with bilateral pars-planitis, commenced on oral prednisolone and referred for paediatric rheumatology and tertiary ophthalmology assessment. The headaches improved following commencement on steroids.

Positive findings on systems review included occasional oral ulcers, 1—2 times-per-month. They started about 1-month prior to the onset of her chickenpox and continued for the following 3—4 months. She denied any history of genital-ulcers or skin-rash. She had new-onset muscle soreness and tiredness after activity. She also described non-specific abdominal pain since having chickenpox, but no associated change in bowel habit.

Examination revealed normal skin, hair, nail and joint examination. She had a soft systolic murmur (echocardiogram normal). She had RUQ tenderness on abdominal palpation (abdominal-USS – spleen upper limit of normal. Nil else). Relevant blood and stool samples were sent as part of a uveitis work-up panel.

The paediatric ophthalmologist found right-intermediate uveitis with vitreitis and peripheral retinal changes in keeping with a peripheral exudative detachment. Similar changes were seen in the peripheral retina of the left eye. The impression was of bilateral pan-uveitis with retinal involvement. A subsequent oral fluorescein angiogram showed a widespread retinal vasculitis with some occlusive changes in the right-eye, and a tuft of retinal vascular leakage at the 5-o'clock position in the left eye.

**Case report - Discussion:**

The differential diagnosis for paediatric retinal vasculitis is broad. It includes collagen vascular disorders, Behçet's disease, Eales’ disease, post viral or post vaccination, acquired toxoplasmosis, multiple sclerosis, systemic immunosuppression and Henoch-Schönlein purpura. The ANA, ENAs, dsDNA, ANCA, ACE, Toxoplasma and Lyme serology were all negative for this little girl. Her inflammatory markers were also normal. She was not on any regular medications prior to her illness and had not had any recent vaccinations. She had no significant past medical or family history of note. However, the onset of her symptoms did correspond to her having chickenpox. She was subsequently found to be positive for HLA B51.

In view of the ophthalmology findings and positive HLA B51, the little girl was admitted for an urgent MRI/MRI to exclude neuro- Behçets. This was reported as normal. She was treated with a three-day pulse of IV methylprednisolone and discharged on oral prednisolone 10mg OD (weight 33.4kg).

At present Bechet’s retinal vasculitis remains high in the list of differentials for this little girl; however, currently she does not strictly meet the diagnostic criteria for Bechet’s disease. She has been commenced on a steroid sparing agent, azathioprine. Her follow-up plan is to be reviewed in the joint paediatric rheumatology and ophthalmology clinic in 1 months’ time.

**Case report - Key learning points:**

Retinal vasculitis may occur secondary to a systemic disease or an infectious agent, or as an isolated retinal aetiology. Given that the differentials are vast, a detailed history and examination are important to identify signs and symptoms of systemic disease. Appropriate investigations should be chosen to help narrow the differentials and ensure pathology that could lead to significant morbidity and mortality is not missed.

With a case such as this, close collaboration between the paediatric ophthalmologist and rheumatologist is paramount to ensure the best outcome for the patient.

With regards to treatment, small case series have described a refractory nature of retinal vasculitis in paediatric patients. One study report that almost 80% of patients with paediatric idiopathic uveitis show manifestations of retinal vasculitis, which is associated with a lower probability of inflammation control resulting in a worse visual prognosis.

**Points for discussion:**

- Thoughts on differential diagnosis?
- Thoughts on appropriate treatment when no definitive cause identified. Should we follow the National Behcet’s Disease pathway?
- Expected prognosis for paediatric retinal vasculitis?

